# Frequency of Human papillomavirus in women attending cervical cancer screening program in Chile

**DOI:** 10.1186/s12885-017-3496-x

**Published:** 2017-08-03

**Authors:** Priscilla Brebi, Carmen Gloria Ili, Alejandra Andana, Doris Menzel, Jaime Lopez, Pablo Guzman, Angelica Melo, Kurt Buchegger, Juan C. Roa

**Affiliations:** 1 Laboratorio de Patología Molecular, Departamento Anatomía Patológica, Facultad de Medicina, Universidad de La Frontera, Casilla 54-D, Temuco, Chile; 2 Centro de Excelencia en Medicina Traslacional-Scientific and Technological Bioresource Nucleus (CEMT-BIOREN), Universidad de La Frontera, Casilla 54-D, Temuco, Chile; 3 Department of Pathology, School of Medicine, Pontificia Universidad Católica de Chile, Marcoleta 377, 7TH Floor, Santiago, Chile; 4 Advanced Center for Chronic Diseases (ACCDiS); Millennium Institute on Immunology and Immunotherapy P09-016-F, Santiago, Chile

**Keywords:** *Human papillomavirus*, Cervical intraepithelial neoplasia, Cervical cancer, Polymerase chain reaction, Reverse line blotting assay

## Abstract

**Background:**

*Human papillomavirus* (HPV) is the etiological factor for cervical cancer and its precursor lesions. The characterization of HPV genotypes in preneoplastic lesions and cervical cancer could establishes the effectiveness of vaccination plan in Chilean population. The aim of this study was to determine HPV frequency in a group of women including in a cervical screening program in the public health care system in Chile.

**Methods:**

We analyzed 985 cervical smears samples from women with different histological diagnosis, attending to public health care in Temuco-Chile between 2004 and 2012, to detect HPV genotypes, through PCR followed by reverse line blotting assay.

**Results:**

HPV was found present in 80.8% (*n* = 796) of samples. Only a 5.6% of 985 samples were infected with a low-risk HPV, considering multiple infections. 10.5% (*n* = 8/76) of normal cervical epithelia, 83.5% (*n* = 208/249) and 87.6% (*n* = 557/636) of low and high grade squamous intraepithelial lesions, respectively, and 95.8% (*n* = 23/24) of squamous cervical carcinomas tested positive for HPV. HPV 16 was the most frequent genotype found (Overall 44.9%, *n* = 442/985; SCC: 62.5%, *n* = 15/24). A high variability of HPV types was also found in preneoplastic lesions, whereas there was a selection of genotypes in neoplasia. Also, there was a higher risk of infection with HPV 16 in women ≤26 years and 34–41 years old (*p* < 0.05), meanwhile infections with HPV 16 or HPV 18 have related with cancer development (*p* < 0.01).

**Conclusions:**

These data provide further information about the frequency of HPV genotypes in women with cervical lesions in Chile, and the introduction of new targeted vaccines against a wider spectrum of HPV is suggested.

**Electronic supplementary material:**

The online version of this article (doi:10.1186/s12885-017-3496-x) contains supplementary material, which is available to authorized users.

## Background

*Human papillomavirus* (HPV) is considered the most frequent sexually transmitted disease in the world [1] and the etiological factor for cervical cancer and its precursor lesions [2]. It is known that HPV infection is present in 99.7% of cervical uterine cancer cases [3].

Cervical cancer is the fourth most common cancer in women with an estimated of 528,000 new cases worldwide, and the second most frequent in less developed regions (445,000 cases), according to data from 2012 [4]. Annually, 266,000 women die of cervical cancer, most of them in developing countries. In Chile, in 2012, the incidence rate of cervical cancer was estimated in 12.8 per 100,000 women and mortality rate in 6.0 per 100,000 women, being the sixth cause of death from malignant tumors in this population [5] and the second most frequent cause of death by carcinoma in young women between 15 and 44 years [6–8].

HPV have been described infecting the skin and mucosa. Approximately 40 HPV genotypes can infect the anogenital mucosa. HPV genotypes are classified according to their capacity to induce carcinogenesis in two groups: low oncogenic risk HPVs (LR-HPV) (genotypes 6, 11, 42, 44, 55, 61, 70, 72, 81, 83) and high oncogenic HPVs (HR-HPV) (genotypes 16, 18, 26, 31, 33, 35, 39, 45, 51, 52, 53, 56, 58, 59, 66, 68, 73, 82). HPV 6 and 11 are the most frequent LR-HPV genotypes and are associated with condyloma acuminata. The HR-HPV genotypes 16 and 18 are frequently found in cervical carcinoma and its precancerous lesions [9]. The oncogenic capacity of HPV is given by the viral oncoproteins E6 and E7, which have the ability of inhibit apoptosis and enhance proliferation of the infected cells. Specifically, E6 promotes the degradation of p53, an important tumor suppressor gene, and E7 interacts with Retinoblastoma (Rb) liberating E2F (transcription factor), which stimulates proliferation of epithelial cells [10].

Cervical carcinogenesis starts with preneoplastic lesions such as mild cervical intraepithelial neoplasia (CIN1), classified as low-grade squamous intraepithelial lesions (LSIL), followed by more severe degrees of neoplasia (CIN2 and CIN3) and carcinoma in situ (CIS), which are classified as high-grade squamous intraepithelial lesions (HSIL) [11, 12].

Worldwide, Papanicoulau smear is the screening technique to detect cervical lesions in women and the detection of HPV genotypes is considered complementary to the clinical diagnosis of patients affected with preneoplastic and neoplastic lesions [13]. HPV is present in different percentages in precancerous lesions: it is found in between 59 and 82% of LSIL, while in HSIL the frequency is generally more than 80% [3, 14–16]. The principal methods for HPV detection are PCR (polymerase chain reaction)-based HPV detection systems, where a broad spectrum of HPV types is amplified by consensus primers, followed by detection with type-specific probes [17]. Also, methodologies based in real-time PCR for detection of DNA and mRNA of HPV, are the most accepted detection methodologies in the last years [18].

The frequency of HPV in Chile has been described in only a few studies, in normal [6, 19], precancerous lesions [20, 21], and cervical cancer samples [22, 23]. The present study illustrates the frequency of HPV in a large number of precancerous lesions in light of the importance of finding the principal HPV genotypes infecting preneoplastic lesions in the Chilean population in order to evaluate the effectiveness of HPV vaccination plan in this country to prevent cervical neoplasia.

## Methods

### Collection of clinical samples

The study corresponded to a cross-sectional type. All women attending to public health care to gynecological control were invited to participate and they signed an informed consent, previously approved by Araucanía Sur Health Service Ethics Committee. A total of 985 samples from cervical scrapes (cytobrush) were collected and evaluated for HPV infection. The median age of enrollment was 33 years old (Interquartile range 15 years). The age of participants was divided into four groups: ≤ 26 years old, between 27 and 33 years old, between 34 and 41 years old and ≥42 years old. Normal epithelium samples (*n* = 76) were collected from women who regularly attending to Miraflores public Clinic for gynecological control, and the cervical cytology (Papanicoulau) was found without alterations in current and past controls. Preneoplastic and neoplastic samples were separated into groups according the histological diagnoses (The 1991 Bethesda System [12]) as follows: 249 LSIL, 636 HSIL and 24 squamous cervical carcinomas (SCC). These samples were collected at the Doctor Hernán Henríquez Aravena Hospital in Temuco, Chile (public hospital), between 2004 and 2012, during gynecological examination. The diagnoses of preneoplastic and neoplastic lesions were confirmed by colposcopy-guided biopsy in the Pathology Anatomy and Citology Unit of Hernán Henríquez Aravena Hospital. Molecular test was performed at Molecular Pathology Laboratory, School of Medicine, Universidad de La Frontera.

### HPV genotyping

Cytobrush samples were deposited in tubes with lysis buffer (Tris 0.01 M pH 7.8, EDTA 0.1 M pH 8, SDS 0.5%) for DNA extraction. The integrity of extracted DNA was evaluated by amplification of a fragment (268 bp) of beta-globin gene using GH20 and PCO4 primers and PCR conditions described previously [20]. PCR for L1 fragment for HPV detection was performed using GP5+ and biotin GP6+ primers as described before [24]. Reverse line blot was performed to ascertain the HPV genotype as described [24]. Briefly, modified oligoprobes [24] for 18 HR-HPV (HPV 16, 18, 26, 31, 33, 34, 35, 39, 45, 51, 52, 53, 56, 58, 59, 66, 68, 73, 82) and 18 LR-HPV (6, 11, 40, 42, 43, 44, 54, 55, 57, 61, 70, 71, 72, 81, 83, 84, IS39, CP6108) were used for the analysis. Oligoprobes bound covalently to a membrane (Biodyne C; Pall Bio-Support West Chester, USA) were activated with EDAC 16% (*w*/*v*) (1-ethyl-3-(3-dimethylaminopropyl) carbodiimide, Sigma St Louis, USA), using the miniblotter system (MN 45; Immunetics Boston, US). The PCR GP5+/biotinylated GP6+ products were denaturalized at 96 °C and cooled in ice prior to the hybridization process. These products were added to each miniblotter channel, perpendicularly to the oligoprobe lines. After 1 hour of hybridization the membrane was removed and washed. Then, the membrane was incubated with streptavidin-peroxidase conjugate (Roche, Basel, Switzerland), washed and incubated with ECL fluid (Amersham Biosciences, Piscataway, NJ, USA), exposed to a film (Hyperfilm; Amersham Biosciences, Piscataway, NJ, USA) and developed using standard reagents to detect the hybridization signal.

### Positive controls

HPV viral types were used for positive controls. HPV 16, 18, 31 and 33 corresponded to commercial plasmid clones (American Type Culture Collection, Manassas, VA, USA), the remaining HPV types were provided by Dr. Peter Snijders (VU University Medical Center, Amsterdam, The Netherlands). Negative controls consisted of commercial genomic DNA (Promega, Madison, WI, US) and deionized water.

### Definitions for single and multiple HPV infections

The detection of only one positive HPV genotype signal in the reverse line blot was defined as a single HPV infection. A multiple infection was defined by the presence of a positive signal for two or more genotypes; in these cases, the predominant signals were considered for further analysis.

### Statistical analysis

All data was analyzed by the software IBM SPSS Statistics 20.0 (IBM Corporation, NY, USA).

Age was categorized in four groups (<26, 26–33, 34–41 and ≥42 years old), due to quartiles determination. In order to improve statistical analysis, due to the low frequency of some HPV genotypes, the less frequent genotypes were grouped as HR-HPV (including HPVs 26, 33, 35, 39, 51, 52, 53, 56, 59, 66 and 68) and LR-HPV. The frequency of HPV genotypes was performed through descriptive statistics using as reference group women ≥42 years old, due to the highest frequencies of HPV infection usually are produced in young women (15–25 years old).

To establish the association between age range or lesion grade and HPV single infection we used multinomial logistic regression. For the analysis of multiple infections and their association with lesion degree, binary logistic regression was used considering only LSIL and HSIL, due the absence of multiple infections in normal and SCC cases. Age and lesion degree were considered as the independent variables in each case. In multinomial and binomial logistic regression, we determine the statistical significance, odd ratio and confidence interval. *P*-values <0.05 were considered significant, with 95% of confidence.

## Results

A total of 985 cytobrush samples were genotyped for HPV. The diagnosis of samples was confirmed histologically, and corresponded to 7.7% (*n* = 76) normal epithelia, 25.3% (*n* = 249) LSIL, 64.6% (*n* = 636) HSIL and 2.4% (*n* = 24) SCC. The median age of enrollment was 33 years old (Interquartile range 15 years).

### HPV detection

**Fig. 1:**
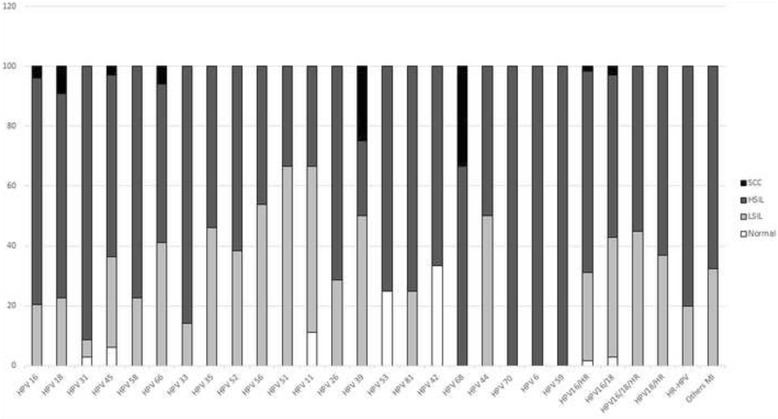
Frequency of HPV genotypes in cytobrush of normal epithelium, preneoplastic and neoplastics lesions of the cervix. HR: High-risk; LR: Low-risk; MI: Multiple Infection

All samples included in this study tested positive for beta-globin PCR (268 bp). After confirmation of DNA integrity, HPV detection using PCR L1 consensus primers was used and 80.8% (*n* = 796) of the analyzed samples were positive for HPV, 78.7% (775) were infected with a HR-HPV and 5.6% (*n* = 55) with a LR-HPV most of them (*n* = 38; 3.9%) combined with a HR-HPV in multiple infections. Considering single (60.3%, *n* = 594) and multiple infections (20.5%, *n* = 202), HPV 16 was the most frequent genotype found (44.9%, *n* = 442) followed by HPV 18 (12.0%, *n* = 118) (Table 1; Fig. 1; Additional file 1: Data S1).

**Table 1:** HPV Infection Frequency in normal epithelium, preneoplastic and neoplastics lesions of the cervix

HPV Status	NORMAL			LSIL			HSIL			SCC		
	n	% Over Total Number of cases	% Over Total Number of SI or MI cases	n	% Over Total Number of cases	% Over Total Number of SI or MI cases	n	% Over Total Number of cases	% Over Total Number of SI or MI cases	n	% Over Total Number of cases	% Over Total Number of SI or MI cases
HPV negative	68	89.5	0.0	41	16.5	0.0	79	12.4	0.0	1	4.2	0.0
Single Infection	*6*	*7.9*	*100.0*	*142*	*57.0*	*100.0*	*425*	*66.8*	*100.0*	*21*	*87.5*	*100.0*
HPV 6	0	0.0	0.0	0	0.0	0.0	1	0.2	0.2	0	0.0	0.0
HPV 11	1	1.3	16.7	5	2.0	3.5	3	0.5	0.7	0	0.0	0.0
HPV 16	0	0.0	0.0	67	26.9	47.2	249	39.2	58.6	13	54.2	61.9
HPV 18	0	0.0	0.0	10	4.0	7.0	30	4.7	7.1	4	16.7	19.0
HPV 26	0	0.0	0.0	2	0.8	1.4	5	0.8	1.2	0	0.0	0.0
HPV 31	1	1.3	16.7	2	0.8	1.4	32	5.0	7.5	0	0.0	0.0
HPV 33	0	0.0	0.0	2	0.8	1.4	12	1.9	2.8	0	0.0	0.0
HPV 35	0	0.0	0.0	6	2.4	4.2	7	1.1	1.6	0	0.0	0.0
HPV 39	0	0.0	0.0	2	0.8	1.4	1	0.2	0.2	1	4.2	4.8
HPV 42	1	1.3	16.7	0	0.0	0.0	2	0.3	0.5	0	0.0	0.0
HPV 44	0	0.0	0.0	1	0.4	0.7	1	0.2	0.2	0	0.0	0.0
HPV 45	2	2.6	33.3	10	4.0	7.0	20	3.1	4.7	1	4.2	4.8
HPV 51	0	0.0	0.0	8	3.2	5.6	4	0.6	0.9	0	0.0	0.0
HPV 52	0	0.0	0.0	5	2.0	3.5	8	1.3	1.9	0	0.0	0.0
HPV 53	1	1.3	16.7	0	0.0	0.0	3	0.5	0.7	0	0.0	0.0
HPV 56	0	0.0	0.0	7	2.8	4.9	6	0.9	1.4	0	0.0	0.0
HPV 58	0	0.0	0.0	7	2.8	4.9	24	3.8	5.6	0	0.0	0.0
HPV 59	0	0.0	0.0	0	0.0	0.0	1	0.2	0.2	0	0.0	0.0
HPV 66	0	0.0	0.0	7	2.8	4.9	9	1.4	2.1	1	4.2	4.8
HPV 68	0	0.0	0.0	0	0.0	0.0	2	0.3	0.5	1	4.2	4.8
HPV 70	0	0.0	0.0	0	0.0	0.0	2	0.3	0.5	0	0.0	0.0
HPV 81	0	0.0	0.0	1	0.4	0.7	3	0.5	0.7	0	0.0	0.0
Multiple Infection	*2*	*2.6*	*100.0*	*66*	*26.5*	*100.0*	*132*	*20.8*	*100.0*	*2*	*8.3*	*100.0*
HPV 16/HRs	1	1.3	50.0	17	6.8	25.8	39	6.1	29.5	1	4.2	50.0
HPV 16/LRs	0	0.0	0.0	6	2.4	9.1	7	1.1	5.3	0	0.0	0.0
HPV 18/HRs	0	0.0	0.0	7	2.8	10.6	12	1.9	9.1	0	0.0	0.0
HPV 16/18	1	1.3	50.0	14	5.6	21.2	19	3.0	14.4	1	4.2	50.0
HPV 16/18/HRs	0	0.0	0.0	9	3.6	13.6	11	1.7	8.3	0	0.0	0.0
HRs-HPV	0	0.0	0.0	6	2.4	9.1	24	3.8	18.2	0	0.0	0.0
HRs/LRs-HPV	0	0.0	0.0	3	1.2	4.5	10	1.6	7.6	0	0.0	0.0
Others MI	0	0.0	0.0	4	1.6	6.1	10	1.6	7.6	0	0.0	0.0
TOTAL	*76*	*100.0*	*-*	*249*	*100.0*	*-*	*636*	*100.0*	*-*	*24*	*100.0*	*-*

*HPV* Human papillomavirus, *LSIL* Low-grade squamous intraepithelial lesions, *HSIL* High-grade squamous intraepithelial lesions, *SCC* Squamous Cervical Carcinoma, *SI* Single Infections, *MI* Multiple Infections, *HR-HPV* High risk Human papillomavirus, *LR-HPV* Low risk Human papillomavirus

### HPV typing in normal epithelia

The median age of patients with normal cytology in this study was 36 years old (Interquartile range 18 years). HPV were positive in 10.5% (*n* = 8/76) of normal cervical epithelia; 7.9% (*n* = 6) in single infections and 2.6% (*n* = 2) in multiple infections. HPV genotypes found in single infections were HPV 45 (2.6%, *n* = 2), HPV 11, 31, 42 and 53 (1.3%, *n* = 1 each). HPV 16 was found only in multiple infections (2.6%, *n* = 2) associated with HPV 18 (1.3%, *n* = 1) and 68 (1.3%, *n* = 1) (Table 1; Fig. 1). Among HPV positive samples, 75% (*n* = 6) of genotypes found in normal samples corresponded to HR-HPV.

### HPV typing in low-grade squamous intraepithelial lesions (LSIL)

Median age of women with LSIL was 32 years old (Interquartile range 19 years). HPV was found in 83.5% (*n* = 208/249) of LSIL patients. Single infections correspond to 57% (*n* = 142) of HPV positive samples and multiple infections to 26.5% (*n* = 66). Including both single and multiple infections, HPV 16 was found in 43% (*n* = 107) of samples, while HPV18 had a frequency of 16.1% (*n* = 40). Others important genotypes found were HPV 45 (8.8%, *n* = 22), HPV 58 (6.0%, *n* = 15) and HPV 52 (5.2%, *n* = 13) (Table 1; Fig. 1). Single infection by LR-HPV was found in only 2.8% (*n* = 7) of single infection (HPV 11, 44 and 81) and 4.4% (*n* = 11) of multiple infections, but always associated with a HR-HPV. HPV 16 associated with any other HPV genotypes was the most frequent combination found in multiple infections (16.1%, *n* = 40).

### HPV typing in high-grade squamous intraepithelial lesions (HSIL)

Median age of women with HSIL was 33 years old (Interquartile range 14 years). The 87.6% (*n* = 557/636) of samples were positive for HPV in HSIL. Single infections corresponded to 66.8% and multiple infections to 20.8%. The most frequents HPV genotypes found in both single and multiple infections were: HPV 16 (50.0%, *n* = 318), HPV 18 (11.3%, *n* = 72), HPV 45 (7.4%, *n* = 47), HPV 31 (7.1%, *n* = 45) and HPV 58 (6.3%, *n* = 40) (Table 1; Fig. 1). Single infection by LR-HPV was found in only 1.9% (*n* = 12) of single infection (HPV 6, 11, 42, 44, 70 and 81) and 3.8% (*n* = 24) of multiple infections, but in 92% (*n* = 22) were associated with a HR-HPV.

### Squamous cervical carcinomas

Median age of women with SCC was 40 years old (Interquartile range 18 years) years. The 95.8% (*n* = 23/24) of cervical cancers were found infected with HPV, mainly by HPV 16 (62.5%, *n* = 15), HPV 18 (25%, *n* = 6), and HPV 66 (8.3% *n* = 2) (Table 1; Fig. 1). Multiple infections represent 8.3% (*n* = 2) of samples, where HPV 16 was found in all cases. There were no found LR-HPV infecting SCC samples.

### HPV association with age and cervical lesion grade

For logistic regression analysis HPV genotyping results were grouped according to single infection and multiple infections HPV genotypes. Single infection genotypes were classified as HPV 16, HPV18, HPV 31, HPV 45, HPV 58, HPV-HR (including HPVs 26, 33, 35, 39, 51, 52, 53, 56, 59, 66 and 68) and HPV-LR (including HPVs 6, 11, 42, 43, 44, 70 and 81). Multiple infections were grouped as HPV16/HRs (*n* = 58), HPV16/LRs (*n* = 13), HPV16/18 (*n* = 35), HPV16/18/HRs (*n* = 20), HPV18/HRs (*n* = 19), HPV-HRs (*n* = 30), HPV-HR/LR (*n* = 13), HPV16/HR/LR (*n* = 4), HPV16/18/LRs (*n* = 2), HPV16/18/HR/LR (*n* = 3), HPV18/LRs (*n* = 1), HPV18/HR/LR (*n* = 2) and HPV-LRs (*n* = 2). To associate age of patients and histopathologic diagnosis of the samples and multiple infections, only were considered groups with higher frequency.

The participants younger than 26 years old (*P* = 0.040; Odd ratio = 1.49; 95% CI: 1.02–2.17) and between 34 and 41 years old (*P* = 0.048; Odd ratio = 1.48; 95% CI: 1.00–2.18) were found to have more risk of infection with HPV 16 among single infections compared to women ≥42 years old (Table 2). No significant relationships were found between age and others HPV single infections. In multiple infections was possible to observe a higher risk of infection with HPV16 combined with a HR-HPV (*P* = 0.032; Odd ratio = 2.50; 95% CI: 1.08–5.77) and HPV18 combined with a HR-HPV (*P* = 0.031; Odd ratio = 3.77; 95% CI: 1.13–12.65) in women ≤26 years old. Meanwhile, individuals between 34 and 41 years old (*P* = 0.029; Odd ratio = 6.1; 95% CI: 1.2–30.99) have more risk of been infected by the combination of HPV 16 and at least one LR-HPV compared to women ≥42 years old. In other hand, women ≤26 years old have less risk of being infected by a combination of HPV16/18 (*P* = 0.036; Odd ratio = 0.34; 95% CI: 0.12–0.93) and others combinations of HR-HPVs (different from HPV16 and 18) (*P* = 0.04; Odd ratio = 0.25; 95% CI: 0.07–0.94) compared to women ≥42 years old. No relationship between age and HPV multiple infections were found.

**Table 2:** HPV infection and its association with age

Age/HPV type	≤26 years			27–33 years			34–41 years			≥42 years		
	p	OR	95% CI	p	OR	95% CI	p	OR	95% CI	p	OR	95% CI
Single Infections												
HPV 16	**0.040**	**1.49**	**1.02–2.17**	0.080	1.41	0.96–2.08	**0.048**	**1.48**	**1.00**–**2.18**	Reference		
HPV 18	0.071	0.38	0.13–1.09	0.975	1.01	0.45**–**2.30	0.461	1.34	0.61**–**2.93	Reference		
HPV 31	0.699	0.83	0.31–2.18	0.979	1.01	0.39**–**2.60	0.907	1.06	0.41**–**2.71	Reference		
HPV 45	0.061	0.33	0.10–1.05	0.846	0.92	0.38–2.20	0.562	0.76	0.30–1.92	Reference		
HPV 58	0.609	1.26	0.52**–**3.04	0.449	0.67	0.23**–**1.90	0.202	0.46	0.14–1.52	Reference		
HR-HPV	0.406	1.29	0.71**–**2.37	0.410	1.30	0.70–2.40	0.122	1.61	0.88–2.93	Reference		
LR-HPV	0.486	0.66	0.21**–**2.11	0.775	1.16	0.42–3.23	0.131	0.30	0.06**–**1.44	Reference		
Multiple Infections												
HPV 16/HRs	**0.032**	**2.50**	**1.08**–**5.77**	0.287	1.65	0.66–4.11	0.100	2.13	0.87**–**5.22	Reference		
HPV 16/LRs	0.875	1.17	0.16**–**8.62	0.697	1.49	0.20–10.99	**0.029**	**6.10**	**1.20–30.99**	Reference		
HPV 18/HRs	**0.031**	**3.77**	**1.13**–**12.65**	0.898	1.11	0.24–5.21	0.368	0.36	0.04**–**3.34	Reference		
HPV 16/18	**0.036**	**0.34**	**0.12–0.93**	0.200	0.53	0.20–1.41	0.070	0.37	0.12–1.08	Reference		
HPV 16/18/HRs	0.134	0.35	0.09**–**1.38	0.692	0.79	0.25**–**2.54	0.269	0.46	0.12–1.82	Reference		
HRs-HPV	**0.040**	**0.25**	**0.07**–**0.94**	0.715	0.83	0.30–2.30	1000	1.00	0.37–2.70	Reference		
HRs/LRs-HPV	0.111	3.81	0.74**–**19.74	0.107	4.01	0.74–21.73	**-**	0.00	0**-**.	Reference		

Values in bold = *p* < 0.05; OR: Odd ratio; *CI* Confidence Interval

*HRs-HPV* High-risk-human papillomavirus. Multiple infections by HPV including 26, 33, 35, 39, 51, 52, 53, 56, 59, 66 and/or 68

*LRs-HPV*: Low-risk-human papillomavirus. Multiple infections by HPV including 6, 11, 42, 44, 55, 70, 72, 81 and/or 83

The analysis of the relationship between histologic grade and the infecting HPV, shows a major risk of single infections by HPV 16 in HSIL (*P* = 0.001; Odd ratio = 1.74; 95% CI = 1.26–2.39) and SCC (*P* = 0.007; Odd ratio = 3.21; 95% CI = 1.37–7.51) compared to LSIL. Also, there is a major risk of single infections by HPV 18 single infection in SCC (*P* = 0.014; Odd ratio = 4.78; 95% CI = 1.38–16.62) and HPV 31 in HSIL (*P* = 0.01; Odd ratio = 6.54; 95% CI = 1.56–27.51) compared to LSIL. In other hand, there is a lower risk of being infected by another HR-HPV in HSIL (*P* = 0.006; Odd ratio = 0.54; 95% CI = 0.350–0.835) compared to LSIL (Table 3). No statistical differences were found in the binomial logistic regression analysis between preneoplastic lesions and multiple infections (Additional file 2: Table S1).

**Table 3:** HPV single infection and its association with cervical lesion grade

HPV genotype	LSIL			HSIL			SCC		
	p	OR	95% CI	p	OR	95% CI	p	OR	95% CI
HPV 16	Reference			**0.001**	**1.74**	**1.26–2.39**	**0.007**	**3.21**	**1.37–7.51**
HPV 18	Reference			0.652	1.18	0.57**–**2.46	**0.014**	**4.78**	**1.38–16.62**
HPV 31	Reference			**0.010**	**6.54**	**1.56**–**27.51**	**-**	0.00	0 **-** .
HPV 45	Reference			0.521	0.78	0.36–1.68	0.971	1.04	0.13–8.48
HPV 58	Reference			0.485	1.36	0.58**–**3.19	**-**	0.00	0 **-** .
HR**-**HPV	Reference			**0.006**	**0.54**	**0.35–0.84**	0.682	0.77	0.22**–**2.70
LR**-**HPV	Reference			0.492	0.72	0.28**–**1.83	**-**	0.00	0 **-** .

Values in bold = *p* < 0.05; OR:Odd ratio; CI Confidence Interval

HPV Human papillomavirus, LSIL Low-grade squamous intraepithelial lesions, HSIL High-grade squamous intraepithelial lesions, SCC Squamous Cervical Carcinoma

HR-HPV High-risk-human papillomavirus, infection by HPV 26, 33, 35, 39, 51, 52, 53, 56, 59, 66 or 68

LR-HPV Low-risk-human papillomavirus, infections by HPV including 6, 11, 42, 44, 55, 70, 72, 81 or 83

## Discussion

Studies around the world have indicated that virtually all cervical cancers contain HPV DNA [2, 22]. HPV positivity varies in different studies according the HPV detection method and type of sample (biopsy or cervical smear) [7, 25, 26]. Due the importance of high-risk HPV infection to the development of cervical cancer, it is necessary to determine HPV genotype distribution in women with or without cervical lesions.

In our study, a total of 985 cytobrush samples (normal, preneoplastic and neoplastics lesions) from women of south of Chile were evaluated for HPV infection. None of these women were vaccinated against HPV. Women with normal cytology have a frequency of 10.5% of infection by HPV. In 2004, Ferreccio et al. in a population-based prevalence study recorded a 14% of HPV infection in women. However, they analyzed general population and some participants had a preneoplastic lesion, therefore they found a higher frequency of HPV infection [6]. Nevertheless, in the same study, Ferreccio found 11.2% of HPV positive in women with normal cytology in Santiago de Chile, results similar to our study [6]. In other hand, we found that majority of HPV positive normal epithelia participants have a HR-HPV infecting. This data also is similar to Ferrecio study in Santiago [6].

The frequency of HPV was elevated in LSIL and HSIL. These percentages are higher than in other studies of HPV frequency in preneoplastic lesions [3, 27]. However, most of HPV frequency investigations, used biopsy samples for HPV detection, in contrast to our study where we used cytobrush samples. It is known that the process of fixation with formalin and paraffin embedding involves DNA fragmentation [28], increasing the number of false-negatives in DNA-based HPV detection. Furthermore, the reverse line blot technique for HPV detection and typing is highly sensitive [22]. About the genotypes found in precancerous samples, studies from Latin-American countries show high frequencies of HPV 16, 18, 45, 31 and 58 in preneoplastics lesions [3, 20, 21, 26, 27, 29], which are correlated with our results.

In cervical cancer, we found a high percentage of HPV infection. Moreover, almost all SCC were positive for HPV and only one sample were tested HPV-negative. The DNA of this sample was tested twice to verify the result. These findings are similar to another study developed by our group, where 94.2% of SCC had the presence of HPV DNA [22].

Almost 30 HPV genotypes were detected in the studied group. The most frequent HPV genotype found was HPV 16, which is usually detected in half of cervical cancer cases and is the most frequent genotype worldwide [7, 22]. Preneoplastic lesions showed the greatest variability in HPV types, while the number of genotypes found in SCC decrease considerably. This could be explained by the immune system clearance in order to eliminate HPV and infected cervical cells. There is a natural regression of preneoplastic lesions in a great number of women infected with HPV [9, 30]. Also, it seems to be a selection of viral genotypes and only the most oncogenic and those with the ability to integrate their genome are capable of cell transformation. This hypothesis is supported by the decrease of the frequency of some HR-HPV genotypes in HSIL and the disappearance of low-risk HPV in SCC [9, 31].

There is agreement regarding the principal HPV genotype found in SCC around the world, namely HPV 16 [9, 22, 32], followed by HPV 18. In our study we found a strong association between these two genotypes and the grade of lesion and SCC. Besides, we found a that HPV 31 is related with a major risk of developing HSIL, which has been observed in several studies [6, 7, 33–35]. More importantly, HPV 16 and/or 18 were found in 56.9% of analyzed samples and genotypes 31, 33, 52 and/or 58 were present in the 16.3%, including single and multiple infection. In 2015, a study performed in Chilean women, showed that 38.8% of women tested positive for HPV were infected with HPV 16 and or 18 [36]. Our results shown a higher frequency of HPV 16 and HPV 18 infections, which is related to the analyzed population, due our study was performed in the Region of La Araucanía, the poorest region of Chile, with the highest native population of the country, and also one of the region with the highest rates of mortality by cervical cancer in Chile [21, 22].

In 2014, Chilean government included, in the national program of immunization, quadrivalent HPV vaccine (HPV 6, 11, 16, 18) for 9 years old girls. This vaccine also has cross-protective efficacy for another HPV genotypes, such as 31, 33, 52 and 58 [37]. Our results indicate that the coverture of HPV vaccine in Chilean population will be wide (approximately 60% of genotypes), and it could prevent a great number of preneoplastics lesion and cervical cancer, diminishing incidence of this pathology. However, these results indicate that almost 40% of SCC could not be prevented by current vaccines.

Otherwise, we found that young women had more risk of being infected by HPV 16 by itself or in association with another HR-HPV including HPV 18. This data suggests an early initiation of sexual relationships and also an early infection by a HR-HPV which could lead to carcinogenesis. Meanwhile, women over 42 years old have more risk of been infected with HPV16 associated with HPV 18 and others types of HR-HPV. Both results could be associated with the description made in others studies, where is observed a high frequency of infection by HPV in young women and a resurgence of the infection in older women [6, 38]. This phenomenon could be explaining due to the beginning of the sexual intercourses in young women, characterized by several sexual partners, followed by period of stable relationship. These results are similar to others shown by Ferreccio et al. [6].

In other hand, PCR-reverse line blotting assay used to genotype HPV was able to detect single and multiple infections easily, because several genotypes can be detected in a single assay, while others methodologies, such as Hybrid Capture 2 assay, only can discriminate between LR and HR genotypes, but it is not capable of indicate the specific genotypes of HPV [39]. Also, there is evidence, that this technique has a high sensitivity, been capable of detect 0.1 fg of HPV [24]. Among the limitations of the study, there was a low collection of normal cytology samples, because the Health care center chose for the participant recruitment was attending mainly women with preneoplastics and neoplastics lesions.

## Conclusions

It is clear that HPV infection continues to be a significant public health problem, particularly in developing countries. There is a wide spectrum of HPV genotypes infecting women around the world and the frequencies of each vary according to the geographic region. Therefore, it is important that genotypes causing cancer in every area be defined. Our results show the distribution of HPV infection through the different alterations in the cervix, finding a genotype selection as the lesion progresses in malignity. The incorporation of quadrivalent HPV vaccine in Chile will potentially diminish HPV frequency in this country, but a great percentage of genotypes will not be covered, evidencing the necessity of new vaccine covering a higher spectrum of HPV genotypes, and given more relevance to HPV detection and genotyping even when patients have been vaccinated for the prevention of cervical cancer.

## Additional files

Additional file 1: Data S1. Specific HPV genotype infecting cervical smears samples from normal epithelium and preneoplastic and neoplastics lesions of the cervix. This data describes for each sample the infecting HPV genotype, according the histology diagnosis. HPV: Human papillomavirus; LSIL: Low-grade squamous intraepithelial lesions; HSIL: High-grade squamous intraepithelial lesions; SCC: Squamous Cervical Carcinoma. (XLS 123 kb)

Additional file 2: Table S1. Multiple HPV infection and its association with preneoplastics lesions. Bivariate Logistic Regression analysis between HPV multiple infection and HSIL. OR:Odd ratio; CI: Confidence Interval; HPV: Human papillomavirus HSIL: High-grade squamous intraepithelial lesions; SCC: Squamous Cervical Carcinoma. * HRs-HPV: High-risk-human papillomavirus, infection by HPV 26, 33, 35, 39, 51, 52, 53, 56, 59, 66 and/or 68. ** LRs-HPV: Low-risk-human papillomavirus, infections by HPV including 6, 11, 42, 44, 55, 70, 72, 81 and/or 83. (XLS 24 kb)

## Abbreviations

CIN: Cervical intraepithelial neoplasia
CIS: Carcinoma in situ
DNA: Deoxyribonucleic acid
EDAC: 1-ethyl-3-(3-dimethylaminopropyl) carbodiimide
HPV: *Human papillomavirus*
HR-HPV: High-risk *Human Papillomavirus*
HSIL: High-grade squamous intraepithelial lesions
LR-HPV: Low-risk *Human Papillomavirus*
LSIL: Low-grade squamous intraepithelial lesions
PCR: Polymerase chain reaction
SCC: *Squamous cervical carcinomas*
